# Brain Cancer in Workers Employed at a Laboratory Research Facility

**DOI:** 10.1371/journal.pone.0113997

**Published:** 2014-12-10

**Authors:** James J. Collins, Thomas John Bender, Eileen M. Bonner, Kenneth M. Bodner, Alisa M. Kreft

**Affiliations:** 1 Health and Human Services, Saginaw Valley State University, University Center, Michigan, United States of America; 2 Health Services/Epidemiology, The Dow Chemical Company, Midland, Michigan, United States of America; 3 Health Services, The Dow Chemical Company, Collegeville, Pennsylvania, United States of America; 4 Epidemiology, Kelly Services, South Lake Tahoe, California, United States of America; 5 EH&S Delivery Expertise, The Dow Chemical Company, Bristol, Pennsylvania, United States of America; Universität Bochum, Germany

## Abstract

**Background:**

An earlier study of research facility workers found more brain cancer deaths than expected, but no workplace exposures were implicated.

**Methods:**

Adding four additional years of vital-status follow-up, we reassessed the risk of death from brain cancer in the same workforce, including 5,284 workers employed between 1963, when the facility opened, and 2007. We compared the work histories of the brain cancer decedents in relationship to when they died and their ages at death.

**Results:**

As in most other studies of laboratory and research workers, we found low rates of total mortality, total cancers, accidents, suicides, and chronic conditions such as heart disease and diabetes. We found no new brain cancer deaths in the four years of additional follow-up. Our best estimate of the brain cancer standardized mortality ratio (SMR) was 1.32 (95% confidence interval [95% CI] 0.66–2.37), but the SMR might have been as high as 1.69. Deaths from benign brain tumors and other non-malignant diseases of the nervous system were at or below expected levels.

**Conclusion:**

With the addition of four more years of follow-up and in the absence of any new brain cancers, the updated estimate of the risk of brain cancer death is smaller than in the original study. There was no consistent pattern among the work histories of decedents that indicated a common causative exposure.

## Introduction

In 2001, a young chemist was diagnosed with brain cancer at a Rohm and Haas research facility in Spring House, Pennsylvania. This event closely followed the death from brain cancer of another former worker at the facility and the publication and press accounts of several brain cancers at the Amoco Research Facility in Illinois [1]–[3]. Based on these concerns, Rohm and Haas initiated a study to determine if employees at this facility had increased risk of brain cancer mortality. This study was done by researchers from the University of Minnesota. Although the study found fewer overall deaths and cancer deaths than expected, there were more brain cancers deaths than expected (14 observed vs. 6.9 expected). However, a nested case-control study found no occupational exposures were related to the excess mortality [4]. Consistent with recommendations from the University of Minnesota and in keeping with the company's comprehensive approach to protecting worker health, The Dow Chemical Company (which acquired Rohm and Haas in 2009) committed to updating the study when the original study was completed.

**Figure 1:**
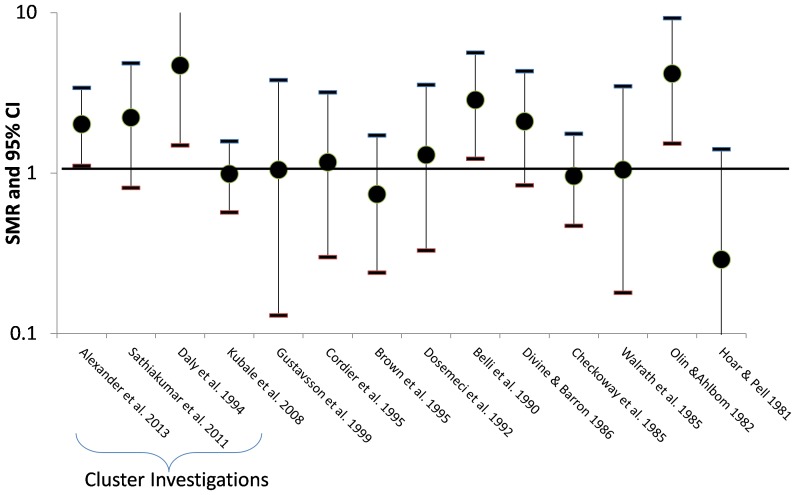
Standardized mortality ratios (SMR) and 95% confidence intervals (95% CI) for studies of laboratory or research workers which report brain cancers.

Several studies have examined cancer mortality or cancer incidence rates among laboratory workers [1]–[26]. Most of these studies reported low overall mortality rates and cancer rates. As shown in Figure 1, there were more brain cancer deaths than expected at several research facilities, including the Spring House and Amoco facilities [3], [4], [10], [15], [25]–[27]. At least three of these studies were cluster investigations prompted by concern that brain cancer risk might be elevated [3], [4], [26]. However, increased cancer rates were reported for other sites besides brain [6]–[8], [10], [11], [14]–[17], [19]–[25], and no study identified an occupational exposure related to the reported cancer excesses. Because of the varied nature of laboratory testing of many substances, exposures in research laboratories such as the Spring House facility are typically more varied, shorter duration, and lower intensity than exposures received by workers in chemical production facilities [28].

## Methods

The original study included 5,284 workers who had ever worked at the Spring House facility. The workers, identified from work records and personnel files, were employed at the facility between its opening in 1963 and the original study end date, December 31, 2007. The work records and personnel files provided names, Social Security Numbers, and work history with start and stop dates for each job for each worker. Since these work records and personnel files were automated computer files, we assumed they were complete. We extended the vital status follow-up to the end of 2011, using sources that included the National Death Index (NDI), the Social Security Death Index, and TransUnion, a credit bureau. The 948 workers terminated or lost to follow-up prior to 1979 were searched first through TransUnion using Social Security Number and name to obtain date of last known address on 869 (92%). From the TransUnion search, there were 211 workers who were either lost to follow-up (79 workers) or had a last known address prior to the study end date (132 workers). These 211 workers were submitted to the Social Security Death Index searching on name, date of birth, and Social Security Number. This search yielded three deaths and dates of death. For 1979 onward, we relied on the National Death Index for deaths and dates of death. We also submitted 132 workers lost to follow-up after these searches to TransUnion and located 75 (57%). We relied on cause of death codes from NDI when an exact match was identified. For less certain NDI matches and for deaths occurring before 1979, we requested death certificates from the state of death. Causes of death on death certificates were coded by a nosologist according to the revision of the International Classification of Diseases (ICD) in effect when the death occurred. Workers accrued person-years at risk from their facility start date until the earliest of the death date, the date lost to follow-up, or 2011. Investigators from the original study provided vital status as of 2007 for all workers. Although information from the original investigators' vital status information was provided, the present study conducted the vital status update without previous knowledge of the original cause or location of death. The study conduct was pursuant to review and oversight by IRB0007144, the Dow Human Studies Review Board in Midland, Michigan, USA. A consent waiver was granted because the study subject records were anonymized and de-identified prior to analysis.

Alexander et al. gathered additional information, not available from NDI or death certificates, to categorize and count brain cancers [4]. Brain tumors on the death certificate can be coded as malignant (ICD-10 C47, C70-72), benign (ICD-10 D32-33, D43), or unspecified as to benign or malignant (ICD-10 C43). If a physician lists only the words “brain tumor” on the death certificate, it is coded as unspecified as to benign or malignant. By obtaining diagnostic or treatment records from medical providers for workers whose cause of death was unspecified brain tumor, Alexander et al. were able to reclassify three deaths as malignant primary brain cancers [4]. In our update, we found no additional primary brain cancers or unspecified brain tumors, and so there was no need for obtaining additional information from medical providers. The exposure assessment done by Alexander et al. for the nested case control study done previously on these workers was extensive (3, 34). Given that no additional brain cancer deaths were observed in our update, we saw no reason to conduct an additional nested case control using the exposure assessment of Alexander et al.

In our study update, the NDI and death certificates were our exclusive sources of cause of death information. As in the original study [4], the 10th revision ICD codes C47 and C70–72 were considered brain cancers along with the corresponding codes from the preceding 7th, 8th, and 9th revisions. We separately reported results for benign brain tumors, mental disorders, and other diseases of the nervous system along with all other causes of death.

We used the Occupational Cohort Mortality Analysis Program (OCMAP) to calculate cause-specific Standardized Mortality Ratios (SMRs) and 95% confidence intervals [29] whereas Alexander et al. used the Life Table Analysis System (LTAS) [30]. Owing to this difference in software, our study and the original study also relied on slightly different comparison rate files. There were some minor differences in classification of some types of unspecified brain tumors in the two rate files across the various revisions of the ICD. To assure comparability in the software and comparison populations, we attempted to replicate the observed and expected numbers of brain cancer deaths for the original study's follow-up period.

In the present study, cause-specific mortality rates for the United States and Pennsylvania were used for comparison and for adjustment for differences in age and sex distribution, time interval, and time since last exposure. We estimated brain cancer risk for four subintervals of the overall follow-up period (i.e., 1963–1980, 1981–1990, 1991–2000, and 2001–2011) to examine how the apparent risk might have varied over time. We also reviewed the work histories, death dates, and age at death for the brain cancer decedents to assess temporal clustering, consistency of latency, and the plausibility of a common etiologic occupational exposure.

## Results

Table 1 compares vital status follow-up for the current study with the original study. The update added 18,688 person years (144,976–126,288) and 139 additional deaths (625–486) to the original study. The percentage of workers lost to follow-up in our updated study was 1.5% (81/5,284). These 81 workers included 4 known foreign or war deaths. We lacked death certificates for two workers known to be deceased through the NDI. However, we were unable to obtain copies of the two certificates from the states where the death occurred.

**Table 1:** Cohort size, follow-up, and results of vital status tracing in original study (Alexander et al.) and current update.

	Original	Update
Cohort size (number of workers)	5,284	5,284
Follow-up		
End date	12-31-2007	12-31-2011
Person-years	126,288	144,976
Vital status tracing		
Alive	4,798 (90.8%)	4,656 (88.1%)
Dead	486 (9.2%)	625 (11.9%)
(+) Death certificate	Not reported	623
(−) Death certificate	Not reported	2
Lost to follow-up or censored	Not reported	81* (1.5%)

^*^This category includes 77 persons lost to follow-up (vital status unknown) and 4 foreign or war deaths. Follow-up for decedents was censored at date of death.

SMRs and 95% confidence intervals based on US and Pennsylvania comparison populations in Table 2 are similar, and so we discuss only the Pennsylvania results. We observed a large deficit for all causes of death combined (SMR = 0.54, 95% CI = 0.49–0.58). This deficit reflects deficits for all cancers combined (SMR = 0.70, 95% CI = 0.61–0.80), diabetes (SMR = 0.23, 95% CI = 0.09–0.46), diseases of the nervous system (SMR = 0.61, 95% CI = 0.36–0.97), cerebrovascular disease (SMR = 0.56, 95% CI = 0.37–0.81), heart disease (SMR = 0.49, 95% CI = 0.42–0.58), non-malignant respiratory disease (SMR = 0.41, 95% CI = 0.29–0.57), cirrhosis of the liver (SMR = 0.18, 95% CI = 0.05–0.47), nephritis and nephrosis (SMR = 0.41, 95% CI = 0.18–0.82), and all external causes of death (SMR = 0.43, 95% CI = 0.31–0.57). The deficit in all cancers combined largely owes to a deficit in lung cancer (SMR = 0.49, 95% CI = 0.36–0.65). We observed more deaths from malignant primary cancer of the brain (or central nervous system) than expected, but the SMR is modestly elevated and imprecise (SMR = 1.32, 95% CI = 0.66–2.37). The SMR for benign brain tumors was 0.87 (95%CI = 0.11–3.17). There was a deficit of non-malignant disease of the nervous system (SMR = 0.61, 95% CI = 0.36–0.97), which included Alzheimer's disease (SMR = 0.67, 95% CI = 0.25–1.49), Parkinson's disease (SMR = 0.53, 95% CI = 0.11–1.54), anterior horn cell disease (SMR = 0.32, 95% CI = 0.01–1.81), and epilepsy (SMR = 0.0, 95% CI = 0.0–4.12).

**Table 2:** Standardized mortality ratio (SMR) and 95% confidence interval (CI) for selected causes of death for all workers based on United States and Pennsylvania comparison populations.

Cause of death (ICD 10th revision codes)§	Observed Deaths	United States	Pennsylvania
		SMR (95% CI)	SMR (95% CI)
All causes (A00-Y89)	625	0.55(0.51–0.60)*	0.54(0.49–0.58)*
All cancers (C00-C97)	225	0.74(0.65–0.84)*	0.70(0.61–0.80)*
Buccal cavity & pharynx (C00-C14)	2	0.32(0.04–1.16)	0.33(0.04–1.20)
Digestive organs & peritoneum (C15-C26, C48)	58	0.79(0.60–1.02)	0.73 (0.56–0.95)*
Esophagus (C15)	4	0.42(0.11–1.07)	0.36 (0.10–0.93)*
Stomach (C16)	8	1.07(0.46–2.11)	1.03 (0.44–2.03)
Large intestine (C18)	20	0.87(0.53–1.34)	0.78 (0.47–1.20)
Rectum (C20–C21)	5	1.11(0.36–2.59)	1.03 (0.33–2.40)
Biliary passages & liver (C22,C24)	7	0.72(0.29–1.48)	0.74 (0.30–1.52)
Pancreas (C25)	11	0.68(0.34–1.21)	0.65 (0.32–1.16)
Other digestive (C17,C19,C23,C26,C48)	3	1.03(0.21–3.02)	0.94 (0.20–2.76)
Respiratory system (C30–C39)	50	0.49(0.36–0.65)*	0.48 (0.36–0.63)*
Bronchus, trachea, lung (C33–C34)	49	0.50(0.37–0.66)*	0.49 (0.36–0.65)
Mesothelioma (C45) 3	3	2.77(0.57–8.09)	2.19 (0.45–6.39)
Breast (C50)	12	1.16(0.60–2.03)	1.10 (0.57–1.91)
All uterine (C53–C55) (females only)	3	1.13 (0.23–3.30)	1.06 (0.22–3.08)
Other female genital (C51–C52, C56–C58)	2	0.63(0.08–2.27)	0.58 (0.07–2.09)
Prostate (C61)	22	1.20(0.75–1.81)	1.16 (0.73–1.76)
Kidney (C64–C65)	6	0.76(0.28–1.65)	0.75 (0.28–1.64)
Bladder and other urinary (C66–C68)	3	0.42 (0.09–1.21)	0.38 (0.08–1.11)
Malignant melanoma (C43)	7	1.19 (0.48–2.45)	1.19 (0.48–2.46)
Central nervous system (C70–72)	11	1.24 (0.62–2.22)	1.32 (0.66–2.37)
Thyroid & other endocrine glands (C73–C75)	2	1.99 (0.24–7.20)	2.04 (0.25–7.35)
All lymphatic & hematopoietic tissue (C81–C96)	27	0.90 (0.60–1.32)	0.86 (0.57–1.26)
Non-Hodgkin lymphoma (C82, C83.0–C83.8, C84, C85.1–C85.9) 2	12	1.04 (0.54–1.81)	0.98 (0.51–1.71)
Leukemia (C91–C95)	9	0.78 (0.36–1.49)	0.75 (0.34–1.42)
Total lymphoid leukemia (C91) 1	0	0.00 (0.00–1.22)	0.00 (0.00–1.19)
Total myeloid leukemia (C92) 1	5	0.89 (0.29–2.07)	0.86 (0.28–2.01)
Acute myeloid leukemia (C92.0) 1	4	0.96 (0.26–2.46)	0.91 (0.25–2.33)
All other leukemia (C93–C95) 1	4	1.54 (0.42–3.95)	1.39 (0.38–3.55)
All other lymphopoietic (C88,C90,C96) 2	5	0.91 (0.30–2.13)	0.91 (0.29–2.11)
All other malignant neoplasms (C44, C46–C47, C76–C79, C80, C97)	19	0.80 (0.48–1.25)	0.66 (0.40–1.04)
Benign brain & CNS tumors (D32–D33, D43)	2	1.08 (0.13–3.92)	0.88 (0.11–3.17)
Diabetes mellitus (E10–E14)	7	0.23 (0.09–0.48)*	0.23 (0.09–0.46)*
Mental disorders (F1–F99)	15	0.77 (0.43–1.26)	0.93 (0.52–1.54)
All diseases of the nervous system (G00–G99)	18	0.60 (0.36–0.95)*	0.61 (0.36–0.97)*
Alzheimer's disease (G30)	6	0.56 (0.21–1.22)	0.69 (0.25–1.49)
Parkinson's disease (G20)	3	0.53 (0.11–1.54)	0.51 (0.10–1.48)
Anterior horn cell disease (G12.29)	1	0.32 (0.01–1.81)	0.32 (0.01–1.80)
Epilepsy (G40)	0	0.00 (0.00–4.12)	0.00 (0.00–4.19)
Cerebrovascular disease (I60–I69)	28	0.56 (0.37–0.81)*	0.56 (0.37–0.81)*
All heart disease (I00–I02, I05–I09, I11, I13–I14, I20–I28, I30–I52)	167	0.53 (0.45–0.62)*	0.49 (0.42–0.58)*
Rheumatic (I00–I02, I05–I09)	1	0.43 (0.01–2.37)	0.35 (0.01–1.93)
Ischemic heart disease (I20–I25)	114	0.50 (0.42–0.60)*	0.49 (0.40–0.58)*
Chronic disease of endocardium & other myocardial insufficiency (I33–I41)	8	0.78 (0.34–1.53)	0.63 (0.27–1.23)
Hypertension with heart disease (I11, I13)	3	0.24 (0.05–0.70)*	0.34 (0.07–0.99)*
All other heart disease (I26–I28, I30–I32, I42–I43, I44–I52)	41	0.64 (0.46–0.87)*	0.52 (0.37–0.71)*
Non-malignant respiratory disease (J00–J99)	36	0.41 (0.28–0.56)*	0.41 (0.29–0.57)*
Influenza & pneumonia (J10–J18)	10	0.46 (0.22–0.85)*	0.49 (0.24–0.91)*
Bronchitis, emphysema, & asthma (J40–J46)	10	0.25 (0.12–0.46)*	0.29 (0.14–0.53)*
Bronchitis (J40–42, J44)	9	0.30 (0.14–0.57)*	0.34 (0.15–0.64)*
Emphysema (J43)	0	0.00 (0.00–0.47)*	0.00 (0.00–0.59)*
Asthma (J45–J46)	1	0.54 (0.01–3.01)	0.60 (0.02–3.35)
Other non-malignant respiratory disease (J00–J06, J20–J22, J30–J39, J47, J60–J70, J80–J86, J90–J99)	16	0.58 (0.33–0.94)*	0.50 (0.28–0.81)*
Ulcer of stomach & duodenum (K25–K27)	1	0.46 (0.01–2.56)	0.57 (0.01–3.19)
Cirrhosis of liver (K70–K74)	4	0.16 (0.04–0.40)*	0.18 (0.05–0.47)*
Nephritis & nephrosis (N00–N29)	8	0.55 (0.24–1.08)	0.41 (0.18–0.82)*
All external causes of death (V01–Y89)	47	0.40 (0.29–0.53)*	0.43 (0.31–0.57)*
Accidents (V01–X59)	26	0.37 (0.24–0.54)*	0.40 (0.26–0.58)*
Motor vehicle accidents (W00–X59)	12	0.37 (0.19–0.64)*	0.45 (0.23–0.78)*
All other accidents (W00–X59)	14	0.38 (0.21–0.63)*	0.36 (0.20–0.60)*
Suicide (W00–X59)	18	0.64 (0.38–1.01)	0.66 (0.39–1.05)
Homicide & other external (X08–Y36, Y40–Y89)	3	0.15 (0.03–0.45)*	0.17 (0.04–0.50)*
All other causes (A00–A09, A20–B19, B25–B99, D00–D09, D37–D42, D44–D45, D47–D89, E00–E07, E15–H99, I70–I99, K00–K23, K28–K67, K71–K73, K75–K93, L00–M99, N30–R99, U01–U99)	54	0.53 (0.40–0.69)*	0.46 (0.34–0.60)
*Unknown causes*	2		
*Number of persons at risk*	5,284		
*Person-years*	144,976		

* Statistically significant at 5% level.

§ Deaths were coded to the ICD revision in force at time of death.

1 Disease classifications not introduced until the 8^th^ Revision of the ICD.

2 Disease classifications not introduced until the 6^th^ Revision of the ICD.

3 Disease classifications not introduced until the 10^th^ Revision of the ICD.

We compared the expected number of brain cancer deaths computed by the two software packages and the two sets of comparison rates (Table 3). The expected number of brain cancer deaths in the original study was 6.9 as compared to our calculation of 6.8 for the same follow-up period (1963–2007). We identified the same 11 malignant primary brain cancer deaths that were reported in the original study. Thus, the SMRs and confidence intervals are very similar for both studies. With no additional brain cancer deaths detected during the extended follow-up (2008–2011), the SMR is now 1.32 (95% CI = 0.66–2.37). Alternatively, we also present the same calculations with a more accurate count of brain cancer cases for the study population, which is based on the additional diagnostic and treatment information collected in the original study. If 14 decedents were counted as brain cancer deaths, then the SMR estimate would be 1.69 (95% CI = 0.96–2.76).

**Table 3:** Observed (Obs) and expected (Exp) number of malignant primary brain cancer deaths, standardized mortality ratio (SMR), and 95% confidence interval (95% CI) in original study (Alexander et al.) and current update, relying only on NDI and death certificates for cause of death information or relying on additional information not included on death certificates, as computed for follow-up through 2007 or 2011.

		Only NDI and death certificates			Additional information		
	Follow-up end date	Obs	Exp	SMR (95% CI)	Obs	Exp	SMR (95% CI)
Original*	2007	11	6.9	1.59 (0.84–2.77)†	14	6.9	2.02 (1.11–3.40)
Update‡	2007	11	6.8	1.60 (0.81–2.92)	14	6.8	2.06 (1.17–3.37)§
	2011	11	8.3	1.32 (0.66–2.37)	14	8.3	1.69 (0.96–2.76)§

^*^Computed using Life Table Analysis System (LTAS).

† We present these values for the convenience of the reader. Alexander et al. did not present an analysis with this observed number, which excludes three deaths with incomplete or modified death certificate information.

‡ Computed using Occupational Cohort Mortality Analysis Program (OCMAP).

§ We present these values for the convenience of the reader. Relying on additional information not included on death certificates provides a more accurate count of deaths in the study cohort, but this approach introduces an information bias relative to the comparison population. We do not consider the ratio of these observed and expected numbers to constitute valid SMR estimates.

Observed brain cancer deaths exceeded expected numbers in each of the three intervals before 2000. However, observed brain cancer deaths were fewer than expected (3 observed versus 4.1 expected, SMR = 0.73, 95% CI = 0.15–2.14) during the final interval (2001–2011) (data not shown).

**Figure 2:**
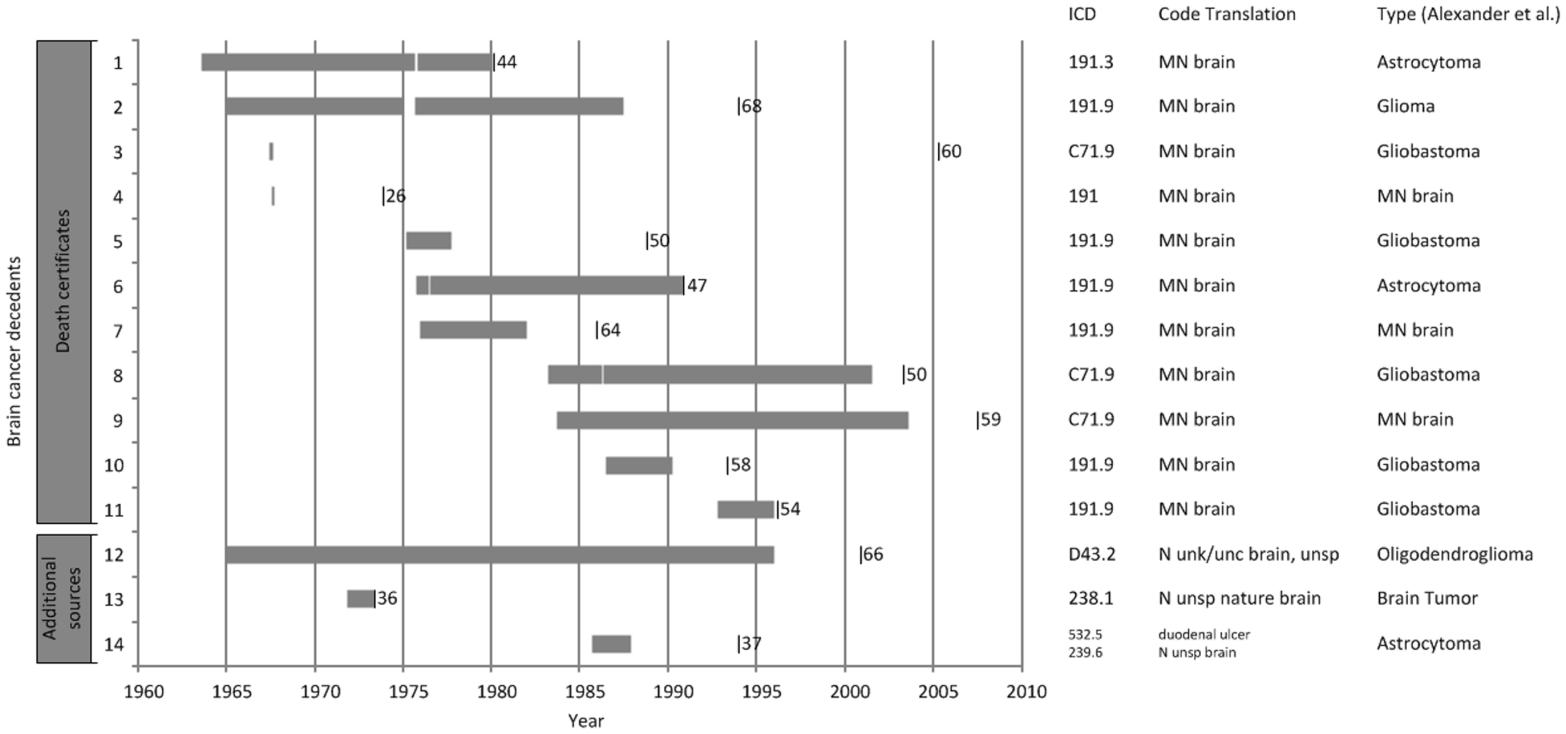
Years worked at the Spring House facility, death date, and age at death for workers identified as having died of brain cancer relying only on NDI and death certificates for cause of death information or relying on additional information not included on death certificates. Gray bars represent years worked at the Spring House facility. The vertical black lines indicate the death date. The numbers specify age at death. The International Classification of Disease (ICD) codes provided were provided by the National Death Index (NDI) or coded by a nosologist according to the ICD revision in effect when the death occurred. ICD code translations are provided along with the type of brain cancer as determined by Alexander et al. in the original Spring House mortality study.

We present a timeline displaying years worked at the Spring House facility, death date, age at death, and cause of death for brain cancer decedents in Figure 2. Among the 14 decedents identified in the original study, duration of employment varied widely with 7 working <5 years and 6 working>10 years. Among short-term workers, there was minimal overlap in the years worked at the facility. Death dates were distributed evenly over four decades. Age at death spanned a wide range (26–68 years).

## Discussion

The results of this update are consistent with the results of the original study. There were still more brain cancer deaths than expected. However, with the addition of four years of follow-up and in the absence of any new brain cancer deaths, the risk estimate for brain cancer death through 2011 is smaller than estimated through 2007. As in most other studies of laboratory and research workers referenced earlier, we found low rates of total mortality, total cancers, accidents, suicides, and chronic conditions such as heart disease and diabetes. While the healthy worker effect is likely responsible for part of these low death rates, the low rates of smoking related deaths in this workforce indicates low smoking rates among these workers. Observed death from lung cancer, heart disease and non-malignant respiratory disease were half the expected levels. We also observed no increased risk in benign brain tumors or in other diseases of the nervous system, including Alzheimer's disease, Parkinson's disease, anterior horn cell disease, and epilepsy. Our ability to replicate the findings of the original study for the same follow-up period is reassuring that both studies were done well.

Brain cancer has been the subject of cluster investigations among laboratory or research workers at two non-Rohm and Haas facilities as well as Spring House [3], [4], [26]. At each facility, previous investigators expended considerable effort identifying all potential brain cancer deaths. Like Alexander et al. did with the present study population [4], Daly et al. investigated a research facility in Ireland [26] and attempted reclassifying deaths coded as “unspecified as to benign or malignant” with additional information not available on the death certificate. Whereas Alexander et al. reclassified 3 deaths, Daly et al. reclassified 1 death. This reclassification provided a count of malignant primary brain cancer that more accurately reflected the burden of disease in the study population. However, this approach may inflate the risk estimate since there was no corresponding adjustment for the general population rates, which were used to compute the expected number of deaths [26], [31]. Given that cluster investigations often occur with input from the study population, reclassification may be justified to assure stakeholders that no brain cancer deaths have been overlooked. However, an accounting of cancer deaths that maintains comparability of cause of death ascertainment for both the study population and the comparison population is likely to provide a more accurate risk estimate.

There is no evidence to support an association between occupational exposure and excess brain cancer mortality as demonstrated in the nested case control study done by Alexander et al. (4,33) Alexander et al. employed a nested case-control study design and used available exposure monitoring date to evaluate five chemical categories: acrylates, bis(chloromethyl) ether, chloromethyl ether, isothiazolones, and nitrosoamines [4], [32]. None of these exposures were related to brain cancer risk nor was work history in general. We wanted to evaluate if there might be any indication of a shared occupational cause – perhaps an unrecognized hazard that was not the subject of exposure monitoring – that might have been responsible for brain cancer developing in all of these workers [33]. If the shared exposure were limited in time, then we might expect to find evidence of temporal clustering of death dates, but instead we observed deaths spanning many years. As such, a common hazardous exposure would have needed to be present for decades, which is unlikely in a workplace characterized by the ever changing exposures of a research facility. Likewise, if a shared exposure were responsible, then there might be some consistency of latency between beginning work and the death date, but instead decedents are evenly split into groups with few and many years worked. The specific types of brain cancer also show no consistency.

Brian cancer clusters are among the more commonly reported and investigated disease clusters [34]. Brain cancer is a devastating disease, with high age-specific incidence rates among working age adults, a low survival rate, and survivorship commonly marked by permanent disability. Workers at a facility like Spring House are highly educated and have comprehensive health insurance, which may contribute to increased diagnostic sensitivity for the disease [35], [36]. When the disease strikes currently employed or recently retired workers, it tends to receive widespread recognition among current workers, as was true at this facility. Both studies of the Spring House facility identified more brain cancer deaths than expected, but the increase in risk was modest and not associated with workplace exposures. With the addition of four more years of follow-up and in the absence of any new brain cancer deaths, the updated estimate of the risk of brain cancer death is smaller than in the original study. Based on available information, the excess brain cancer mortality among Spring House workers is unlikely to be due to a workplace explanation and might be the result of chance or confounding by non-occupational risk factors.
